# Primary malignant melanoma of the esophagus: a rare and aggressive disease

**DOI:** 10.1186/1477-7819-11-210

**Published:** 2013-08-23

**Authors:** Flávio Hiroshi Ananias Morita, Ulysses Ribeiro, Rubens Antonio Aissar Sallum, Marcos Roberto Tacconi, Flávio Roberto Takeda, Julio Rafael Mariano da Rocha, Giovanna de Sanctis Callegari Ligabó, Evandro Sobrosa de Melo, Wilson Modesto Pollara, Ivan Cecconello

**Affiliations:** 1 Departments of Gastroenterology/Pathology, Instituto do Câncer do Estado de São Paulo - ICESP, University of São Paulo School of Medicine, São Paulo, Brazil

**Keywords:** Primary malignant melanoma of the esophagus, Metastasis, Diagnosis, Surgery, Treatment

## Abstract

Primary malignant melanoma of the esophagus is an uncommon tumor, with approximately 300 cases having been reported thus far. The purpose of this study was to describe a case of a 60 year-old man with a 10 month history of progressive dysphagia and thoracic pain, the investigations of which led to a diagnosis of primary malignant melanoma of the esophagus. The patient underwent a transhiatal esophagectomy with subcarinal lymphadenectomy, and isoperistaltic gastric tube replacement of the esophagus. Nine months after surgery, he developed ischemic colitis, and metastasis in the mesentery was diagnosed. His disease progressed and he died one year after the esophagectomy. A review of the literature was performed.

## Background

Melanoma is more frequently found in sun-exposed areas; however it can appear in other sites including the mucosal surfaces [1]. Primary malignant melanoma of the esophagus is more common in the elderly and the diagnosis is usually made in advanced stages of the disease; it has a worse prognosis than cutaneous melanoma.

Melanocytes are present in small amounts in the squamous epithelium or basal membrane of the esophagus, and can be the precursors of melanocytosis and primary malignant melanoma.

Primary malignant melanoma of the esophagus is characterized by aggressive local invasion and multiple and early metastasis. Despite the availability of multimodal therapies such as radical surgery, chemotherapy, radiotherapy, and immunotherapy, the prognosis is still poor [2-4]. More recently, the prognosis seems to have improved, and studies suggest that this may be associated with tumor early detection.

## Case presentation

A 60 year-old man was hospitalized with a 10 month history of progressive dysphagia and chest pain. He was a former smoker for 50 years (40 cigarettes/day). He denied personal or family history of cancer. On physical examination, no skin, ocular lesions, or palpable lymph nodes were detected.

**Figure 1:**
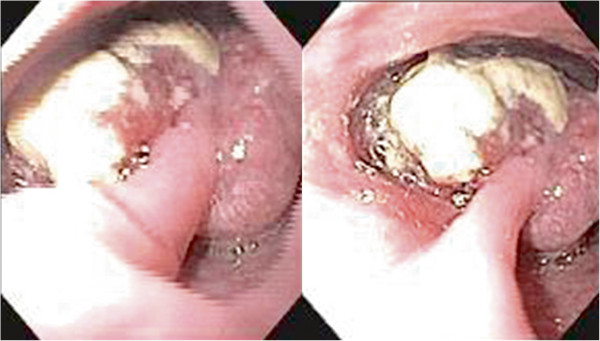
Polypoid tumor measuring 8.0 cm, located in the lower third of the esophagus, which occupied 80% of the esophageal lumen.

**Figure 2:**
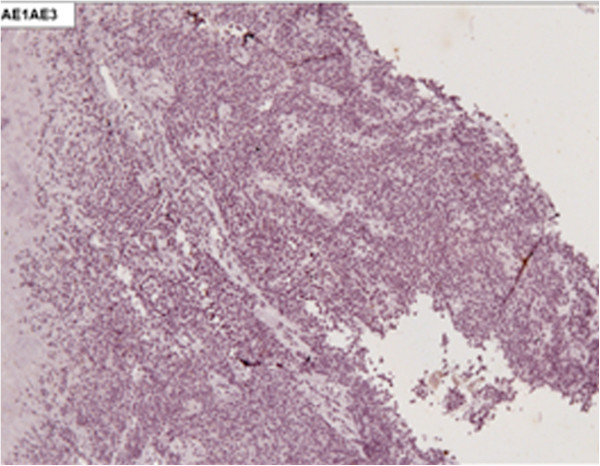
Immunohistochemical staining negative for AE-1/AE-3 (X100).

**Figure 3:**
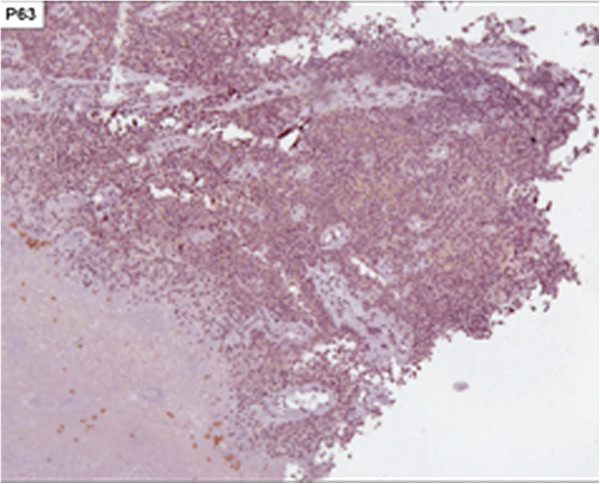
Immunohistochemical staining negative for P63 protein (X100).

**Figure 4:**
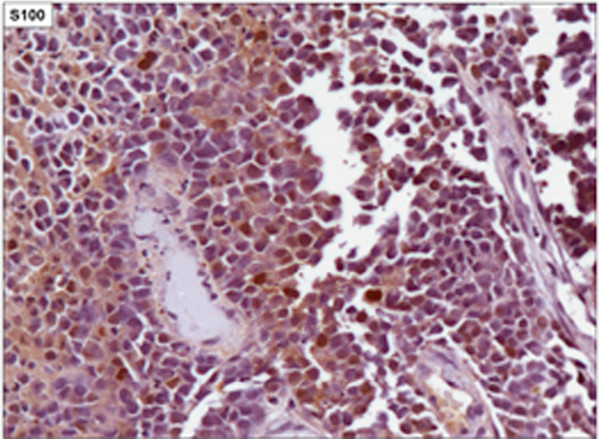
Immunohistochemical staining positive focal for S-100 protein (X400).

**Figure 5:**
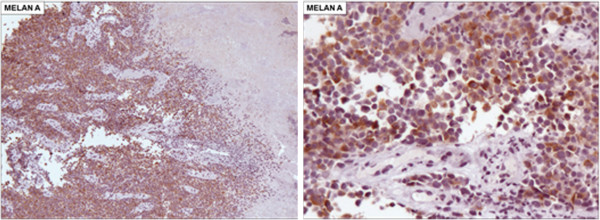
Immunohistochemical staining positive for melanA protein (5A. X100 and 5B. X200).

**Figure 6:**
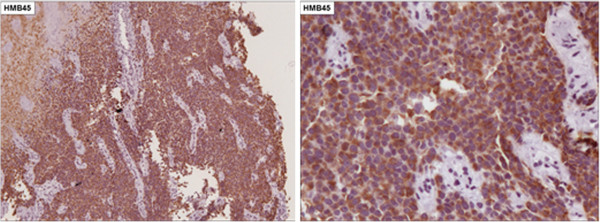
Immunohistochemical staining positive for HMB-45 protein (6A. X100 and 6B. X200).

Upper digestive endoscopy showed an 8.0 cm polypoid tumor, located in the lower third of the esophagus, about 30 cm from the upper dental arch, which occupied 80% of the esophageal lumen (Figure 1). Biopsies with immunohistochemical studies were compatible with malignant melanoma: AE-1/AE-3 negative, P63 protein negative, S-100 protein positive focal, melanA positive and HMB-45 positive (Figures 2, 3, 4, 5 and 6).

**Figure 7:**
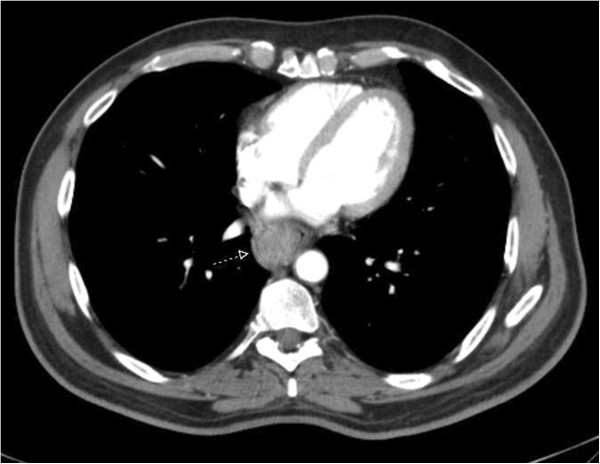
CT scan shows a bulky tumor in the lower third of the esophagus (arrow).

**Figure 8:**
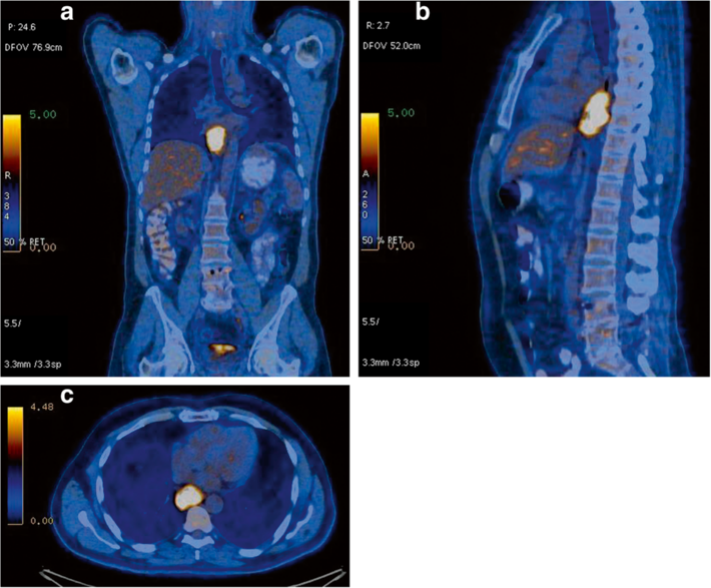
**PET-CT demonstrates FDG uptake only in the esophageal lesion, with no evidence of lymph node or distant metastatic disease.a)** front view; **b)** lateral view; **c)** PET-CT transversal section showing the esophageal tumor uptake.

Computed tomography (CT) was performed for clinical staging which showed a 4.7 × 3.8 × 2.1 cm tumor in the lower third of the esophagus (Figure 7). There were peritracheal lymph nodes measuring 1.4 × 0.9 cm near the esophagogastric junction. Positron emission tomography (PET-CT) showed uptake of fluorine-18-2-fluoro-2-deoxy-D-glucose (FDG) only in the esophageal tumor, with no evidence of lymph node or distant metastatic disease (Figures 8).

The patient underwent transhiatal esophagectomy with subcarinal lymphadenectomy, and isoperistaltic gastric tube replacement of the esophagus. During the operation, no evidence of metastatic disease was observed. Anatomopathologic examination confirmed malignant melanoma of nodular type, measuring 8.5 cm, with infiltration of the muscular layer up to 3.3 cm. There was no lymphatic or venous invasion. There was evidence of perineural invasion, desmoplasmin mild peritumoral, mild intra and peritumoral lymphocytic response, ulceration, and the rate of four mitoses per 10 fields. The margins were free from malignant involvement. There were 26 dissected lymph nodes (11 in the esophageal adventitia, 1 at the esophagogastric junction and 14 at the lesser curvature and celiac trunk) without evidence of malignancy.

The patient’s postoperative recovery was uneventful. Five months after the surgery, a CT scan suggested lymph node recurrences in the left external iliac chain, subcarinal and perigastric nodes. A fine-needle endoscopic-ultrasound-guided aspiration confirmed the recurrence. The molecular analysis for mutation of *c-KIT* was negative. Afterwards, chemotherapy with dacarbazine was started.

**Figure 9:**
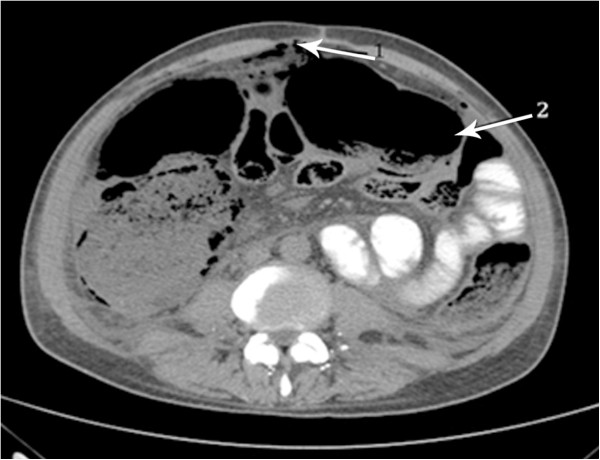
Pneumoperitoneum (arrow 1), gaseous distension of the colon (arrow 2) and the presence of free fluid in the abdominal cavity is shown in the abdominal CT.

**Figure 10:**
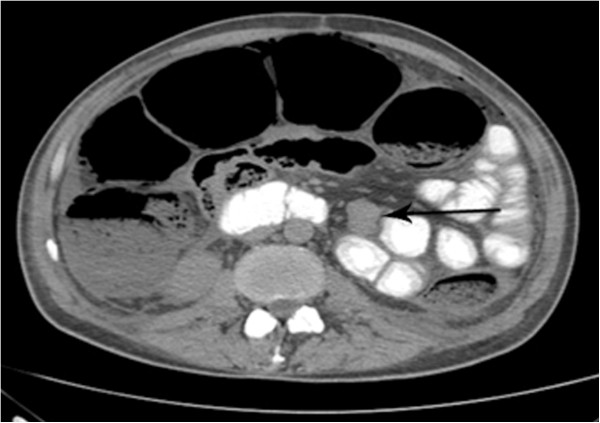
Abdominal CT shows hypodense nodular lesion next to the jejunum in the left flank, measuring 2.5 × 1.8 cm.

Nine months after surgical treatment and the second cycle of chemotherapy, he was admitted to hospital due to abdominal distension and pain. The abdominal CT showed pneumoperitoneum (Figure 9) without evidence of iodine outside the intestinal lumen. The main features were of intestinal gaseous distension of the colon (Figure 9) and the presence of free fluid in the abdominal cavity. There was a hypodense nodular lesion next to the jejunum in the left flank measuring 2.5 × 1.8 cm that appeared to be metastatic disease (Figure 10).

Also observed were left external iliac chain lymphadenopathy, bilateral pleural effusions and a poorly characterized mediastinal mass. An exploratory laparotomy was performed which diagnosed an ischemic colitis, and a lobulated pigmented lesion measuring 2.0 cm, located 40 cm from the Treitz’s angle. Subtotal colectomy and terminal ileostomy were performed. The patient’s recovery was uneventful and he was started on therapy with ipilimumab. However, his disease progressed and he died one year after the first surgical intervention.

### Discussion

The first case of primary malignant melanoma of the esophagus was reported by Baur in 1906. It is believed that in common with those occurring in the skin, melanocytes present in the esophageal epithelium are derived from neural crest tissue from where they migrate during embryogenesis [5,6].

It is a rare disease with estimated frequency of 0.1 to 0.2% of all malignancies in the esophagus, with a total of 337 reported cases up to the year 2011 [7,8].

Up until now, no risk group has been identified. Studies have been showing an average age at diagnosis of 60.5 years, and a male to female prevalence ratio of 2:1 [2], the present case providing an example of this male preponderance. The most common locations are in the lower and middle thirds of the esophagus, as reported in more than 90% of cases in some series. This is due to the fact that there is a greater concentration of melanocytes in this location [6].

Esophageal melanocytosis is a benign condition in which there is proliferation of melanocytes in the basal layer, with consequent increase in the amount of melanin in the mucosa. The immunohistochemistry is positive for melanocytic markers, such as S-100, melanA and HMB-45. Melanocytosis occurs in 25 to 30% of patients with primary malignant melanoma of the esophagus and in 7.7% of subjects without esophageal disease, suggesting this as a predisposing factor for this disease [2].

The most common symptoms of primary malignant melanoma of the esophagus are dysphagia and epigastric pain which are present in 70% of patients, as well as in our case. The occurrence of hematemesis or melena is unusual.

The diagnosis is often difficult, since the symptoms are nonspecific and there is often a low suspicion level due to the rarity of the disease. This is especially so in the absence of evident melanin granules as diagnostic errors may occur because the lesions are not considered as showing clearly differentiated carcinoma. In addition, the differential diagnosis between primary and metastatic melanoma of the esophagus is difficult and some published studies suggest some criteria for differentiation between primary and metastatic disease [9].

Although radiological studies such as esophagography, computed tomography and magnetic resonance imaging can identify and locate the tumor, the diagnosis can only be established by upper digestive endoscopy with biopsy and immunohistochemical studies [10]. During endoscopic examination, primary esophageal melanoma usually appears as a pedunculated polypoid and pigmented tumor, covered by normal mucosa and not ulcerated. Its color varies depending on the amount of melanin, which can be absent (amelanotic melanoma). The presence of other lesions in the esophageal wall, called satellite injuries, indicates intramural metastasis of the melanoma. In addition to the presence of melanocytosis, the diagnosis of melanoma *in situ* can support the diagnosis of the primary disease.

Histological findings consist of pigmented cells that grow wildly and show irregular, fusiform or similar structures to the epithelioid cells. In nearly 20 to 50% of the cases, melanin granules are not detected in the cytoplasm. In these cases, immunohistochemical positive study for S-100 protein, HMB-45, neuron-specific enolase and negative for cytokeratin and CEA confirm the diagnosis of melanoma and exclude carcinoma.

In a review of 10 cases, in order to differentiate between primary and metastatic lesions, of which five had primary malignant melanoma of the esophagus, and the other five, metastatic melanoma to the esophagus, it was observed that in 100% of the cases of metastatic disease, there was a prior history of cutaneous malignant melanoma which was not present in any of the cases in the other group. Most patients with metastatic disease in the esophagus had metastases in other sites, suggesting that metastasis to the esophagus occurs only in a very advanced stage of the disease. The criteria established by this study indicate that primary melanomatous diseases of the esophagus were: the presence of melanoma *in situ*, the presence of a radial growth phase, melanocytosis and a mixture of histology of epithelioid and fusiform cells, in the absence of a history of melanoma at another site [9].

The most frequent site of metastasis is the regional lymph nodes (40 to 80%) such as the periesophageal chain followed by distant lymph nodes and organs including the liver (31%), lungs (18%) and brain (13%). Autopsies of 45 patients showed evidence of metastasis in 78% [11].

The clinical staging can be performed by endoscopic ultrasound, especially in non-advanced tumors, CT, and/or magnetic resonance imaging, and PET.

The seventh edition of the TNM, published by the American Joint Comission on Cancer in 2010, introduced a new classification for melanoma of the upper aerodigestive tract. In this classification T1, T2 and stages 1 and 2 were left out due to the aggressive nature of mucosal melanomas (Tables 1 and 2) [12]. This classification is not widely utilized in the literature and it was mentioned only by Langer *et al*. [13]. Other authors have used the TNM classification similarly for staging of squamous cell carcinoma and adenocarcinoma.

**Table 1:** T classification for melanoma of the upper aerodigestive tract

**T3**	**Epithelium (mucosal disease)**
T4a	Deep soft tissue, cartilage, bone or overlying skin
T4b	Brain, dura, skull base, lower cranial nerves, masticator space, carotid artery, prevertebral space, mediastinal structures, cartilage, skeletal muscle or bone.

AJCC [12].

**Table 2:** TNM classification

**Stage III**	**T3**	**N0**	**M0**
Stage IVA	T4a	N0	M0
	T3-T4a	N1	M0
Stage IVB	T4b	Any N	M0
Stage IVC	Any T	Any N	M1

AJCC [12].

This classification differentiates adequately the prognosis, T3 having a considerably better prognosis than T4, and emphasizes the aggressive nature of melanoma compared to adenocarcinoma and squamous cell carcinoma. The rarity of primary malignant melanoma of the esophagus prevents randomized studies to support therapeutic decisions being carried out. In the absence of prospective randomized controlled studies, the review of case reports points out that total, almost total, or partial esophagectomy, with or without gastrectomy is the preferred surgical treatment in resectable disease patients [3,4,14,15]. Surgical resection is the only modality treatment that influences survival.

Esophagectomy with three-field lymph node dissection (periesophageal, mediastinal and celiac trunk) is the treatment of choice when possible [8]. Surgical margins must be broader than in other esophageal malignancies, as satellite lesions are frequent. However this procedure is associated with high postoperative morbidity and mortality, and it is difficult to determine the cost benefit, since most patients die from disease recurrence in a short period of time even after curative resection, as occurred in this present case [16]. As reported by Adili and Mönig, the resection of isolated metastases even in different locations, can be beneficial for long-term survival [6].

Mukaya *et al*. reported a case of successful surgical treatment for tumor recurrence. In a literature review conducted by the authors (another case), it has been shown that surgical resection, when possible, is the treatment of choice in cases of tumor recurrence [17].

Endoscopic mucosal resection can be performed for tumors restricted to the mucosal layer. It is mainly indicated for patients with associated diseases which discourage surgery. It should not be performed for tumors that invade the submucosal layer or in the presence of metastatic lymph nodes. The degree of tumor invasion and metastatic lymph node involvement can be effectively evaluated using endoscopic ultrasound [17]. A careful histological analysis of the resected segment, ensuring free margins and a rigorous post-procedure follow-up are necessary. Endoscopic laser ablation can be used as palliative treatment in locally advanced tumors.

Other therapeutic modalities such as radiotherapy, chemotherapy and immunotherapy have controversial efficacy. Radiation therapy is indicated for obstructive unresectable tumors as a palliative treatment. Endoscopic metallic stents can also be used for this purpose.

Chemotherapy usually includes dacarbazine, nimustine, vincristine and cisplatin. Dacarbazine confirmed its efficacy for cutaneous malignant melanoma. A randomized controlled trial which compared the Dartmouth regimen (dacarbazine, cisplatin, bischloroethylnitrosourea and tamoxifen), CVD (cisplatin, vindesine and dacarbazine) and BOLD (bleomycin, vincristine, dacarbazine and loumostine) with only dacarbazine, for patients with metastatic melanoma, did not show survival improvement for these associations when compared with isolated dacarbazine [2].

Immunotherapy has evolved considerably. Currently, new therapeutic modalities have appeared in addition to interferon, as vaccines for activating the dendritic cells as well the passive immunotherapy as lymphokine-activating killer cell and monoclonal therapy directed to specific antigens of the melanoma.

Studies with various agents were performed but no gold standard systemic therapy has been found. None of these agents showed increased overall survival, although they have shown some beneficial effect. It is difficult to assess the value of these studies for primary malignant melanoma of the esophagus because these tumors are rare; most of what is applied is the extrapolation of results from studies of melanomas of other sites or from studies providing low grades of evidence.

Molecular analysis could identify targets as an option for additional specific therapy in tumors of a high degree of malignancy. In a series of ten patients with primary malignant melanoma of the esophagus studied by Langer *et al*., the mutation of *c-KIT*, among others was detected in two by molecular analysis, and in all by immunohistochemistry [13]. The detection of this mutation by molecular analysis is relevant, since its presence, although rare, in primary malignant melanoma of the esophagus, may be related to response to therapy with tyrosine kinase inhibitor. In the reported case, the research for molecular analysis of mutation of c-Kit was negative.

The prognosis of malignant melanoma of the esophagus is dreadful [10,16], because in addition to having a highly aggressive biological behavior, most patients are diagnosed late, with the disease at an advanced stage [15]. The improvement in survival of these patients depends on early diagnosis. In a literature review, Bisceglia *et al*. studied the rate of long-term survival adjusted to the T stage. They found that all patients who survived 36 months or more had stage T1, except for one whose staging of the tumor was pT2pN0, therefore, emphasizing the importance of early diagnosis. Sabanathan, in a series of 139 patients, reported an average survival rate of 10 months and a five-year survival after surgical treatment of 4.2% [18]. More recently, Volpin *et al*. published a study with 25 patients, in whom the five-year survival was 37%, and this finding was mainly attributed to early diagnosis.

## Conclusions

Malignant melanoma of the esophagus is a rare and aggressive disease. Its diagnosis is difficult and usually made late, in the advanced stages of the disease, providing a poor prognosis with low long-term survival rates even after appropriate treatment. A better survival rate can be achieved if the diagnosis is made early. The treatment of choice is surgical resection whenever possible, even in cases of recurrent or metastatic disease. Radiotherapy and chemotherapy have not shown benefits in survival rates to date. Other forms of therapy are being studied, but still no benefits in survival rates have been demonstrated. Currently, studies with a high level of evidence are not feasible for implementation, due to the rarity of the disease.

## Consent

The patient consented to the study and publication of this case report and images. A written consent is available for review.

## Abbreviations

CT: Computed tomography; PET-CT: Positron emission tomography; FDG: fluorine-18-2-fluoro-2-deoxy-D-glucose.

## Competing interests

The authors declare that they have no competing interests.

## Authors’ contribution

We declare that FHAM, URJ, RAAS, MRT, FRT, JRMR, ESM, WMP and IC were involved in the care of the patient. GSCL reviewed and photographed the pathology and immunohistochemistry analysis. All authors performed research/study. FHAM and URJ wrote the first draft of the manuscript. All authors read and approved the final manuscript.
